# Deep Learning-Based Versus Threshold-Based Bone Removal for CT Angiography of Head and Neck Arteriovenous Fistulas: A Retrospective Comparison of Image Quality, Key-Structure Display, and Post-Processing Time

**DOI:** 10.3390/diagnostics16203327

**Published:** 2026-10-13

**Authors:** Yiting Wang, Li Tao, Wen Li, Yajing Yang, Xinnan Shen, Meiqi Wang, Zhiwei Zhang

**Affiliations:** Department of Radiology, The First Affiliated Hospital of Chongqing Medical University, No. 1 Youyi Road, Yuzhong District, Chongqing 400016, China; wyt_171728@163.com (Y.W.); tlhunter@163.com (L.T.); liwencqmu@163.com (W.L.); yyj12311016@163.com (Y.Y.); 19562105306@163.com (X.S.); 17361359906@163.com (M.W.)

**Keywords:** deep learning, computed tomography angiography, bone removal, arteriovenous fistula, digital subtraction angiography, image quality, post-processing, head and neck

## Abstract

**Background/Objectives:** Bone overlap at the skull base limits CT angiography (CTA) in head and neck arteriovenous fistulas (hAVFs), and threshold-based bone removal fails where bone and contrast attenuation overlap. Whether deep learning-based removal improves the display of the structures that determine endovascular planning is unknown in a DSA-confirmed cohort. We therefore compared the two methods within the same CTA datasets. **Methods:** In this single-centre retrospective study, 35 patients with DSA-confirmed hAVF underwent head and neck CTA and digital subtraction angiography (DSA) within two weeks of each other, and each CTA dataset was post-processed with both methods on one workstation. Two blinded readers scored the fistula site, feeding artery and draining vein (0 to 2 each) and overall image quality (5-point scale). Scores were compared with paired Wilcoxon signed-rank tests, vessel edge sharpness and post-processing time with paired t tests, and technical visualisation with McNemar’s test. **Results:** Deep learning-based removal displayed the fistula site, feeding artery and draining vein more completely (median total anatomical score 6.00 versus 3.00; *p* < 0.001 for both readers) and overall image quality was higher (5.00 versus 4.00; *p* < 0.001). Objective vessel edge sharpness was concordant (316.47 ± 46.68 versus 273.70 ± 45.66 HU/mm; *p* < 0.001). Technical visualisation was numerically higher (97.1% versus 88.6%) but not significantly so (*p* = 0.248; three discordant pairs), which makes this endpoint inconclusive rather than negative. Post-processing time was shorter (40.56 ± 1.61 s versus 101.70 ± 4.39 s; mean paired difference 61.14 s, 95% CI 59.53 to 62.74 s; paired t test, *p* < 0.001). **Conclusions:** In DSA-confirmed hAVF, deep learning-based bone removal displayed the structures that determine endovascular planning more completely than threshold-based removal and was about one minute faster per case. It does not replace DSA for definitive assessment of fistula architecture, and the apparent advantage at the cavernous sinus is exploratory and requires external validation in larger multicentre cohorts.

## 1. Introduction

An arteriovenous fistula (AVF) is an abnormal direct communication between an artery and a vein without an intervening capillary bed. Intracranial and spinal dural AVFs are classified by location and venous drainage pattern [1]. Head and neck arteriovenous fistulas (hAVFs) are uncommon. They can cause pulsatile tinnitus, headache, ocular congestion and cranial nerve deficits, and they may be complicated by intracranial haemorrhage or hypertensive venous encephalopathy [2,3,4,5,6,7]. These presentations overlap with other high-flow head and neck vascular lesions, so careful imaging differentiation is required [8]. Management depends on identifying the fistula site, the feeding arteries and the draining veins and on recognising high-risk drainage patterns [9,10,11,12]. Digital subtraction angiography (DSA) is the reference standard, but it is invasive and carries a risk of thromboembolic complications [13]. Reliable non-invasive assessment is therefore valuable.

CT angiography (CTA) is widely used to evaluate head and neck vascular disease [14]. At the skull base and in the neck, bone attenuation approximates that of opacified vessels, so bone overlap degrades vessel visualisation. The effect is most pronounced for the thin, tortuous shunting vessels that lie adjacent to bone. Bone removal is therefore a decisive step in CTA post-processing. Conventional threshold-based bone removal relies on fixed or empirically chosen attenuation thresholds; because the attenuation ranges of bone, calcification and contrast-enhanced blood overlap, and because partial volume effects vary locally, it can leave residual bone or erroneously remove vessel voxels. Both failures create pseudo-defects that lower image quality and reader confidence [15]. The failure is the worst at the skull base, the petrous apex and the parasellar region, which are also frequent hAVF locations.

Deep learning has improved medical image segmentation, reconstruction and post-processing [16,17,18,19]. Applied to CTA bone removal, it has been reported to outperform conventional algorithms in bone-removal performance, vessel-branch completeness and vascular integrity in head and neck CTA [20], to shorten reconstruction time [21] and to achieve high skull-segmentation accuracy [22], and it has improved a clinical detection endpoint on brain CTA [23], and image quality in cervical CTA [24]. Deep learning reconstruction has also improved vascular edge sharpness and visualisation of small-calibre external carotid artery branches on head and neck CTA [25]. In all of these studies the endpoint was general image quality or general vessel display, and none anchored the comparison to a DSA reference within a defined disease cohort. None scored the fistula site, the feeding artery and the draining vein, which are the structures that determine whether an hAVF can be treated endovascularly and by which route. It therefore remains unknown whether better bone removal translates into better display of the structures that interventional planning requires. Such processing sits within the broader Internet of Medical Things (IoMT) framework, in which connected medical devices and information systems support data sharing and intelligent image analysis [26]. The question addressed here concerns that AI-based image-processing component rather than networked infrastructure. We therefore compared deep learning-based with threshold-based bone removal in patients with DSA-confirmed hAVF. Each CTA dataset was processed with both methods on the same workstation under identical settings. We assessed subjective image quality, display of the fistula site, feeding artery and draining vein, technical visualisation rate and post-processing time, and we explored whether the effect differed by lesion location. The study evaluates display in known-positive cases, not diagnostic accuracy.

## 2. Materials and Methods

### 2.1. Study Design and Participants

In this single-centre retrospective study, patients were consecutively enrolled between January 2021 and July 2025. All underwent both head and neck CTA and DSA at the First Affiliated Hospital of Chongqing Medical University. Because of the retrospective design, the sample size was determined by the number of eligible consecutive patients during the study period, and no formal a priori sample-size calculation was performed.

**Figure 1:**
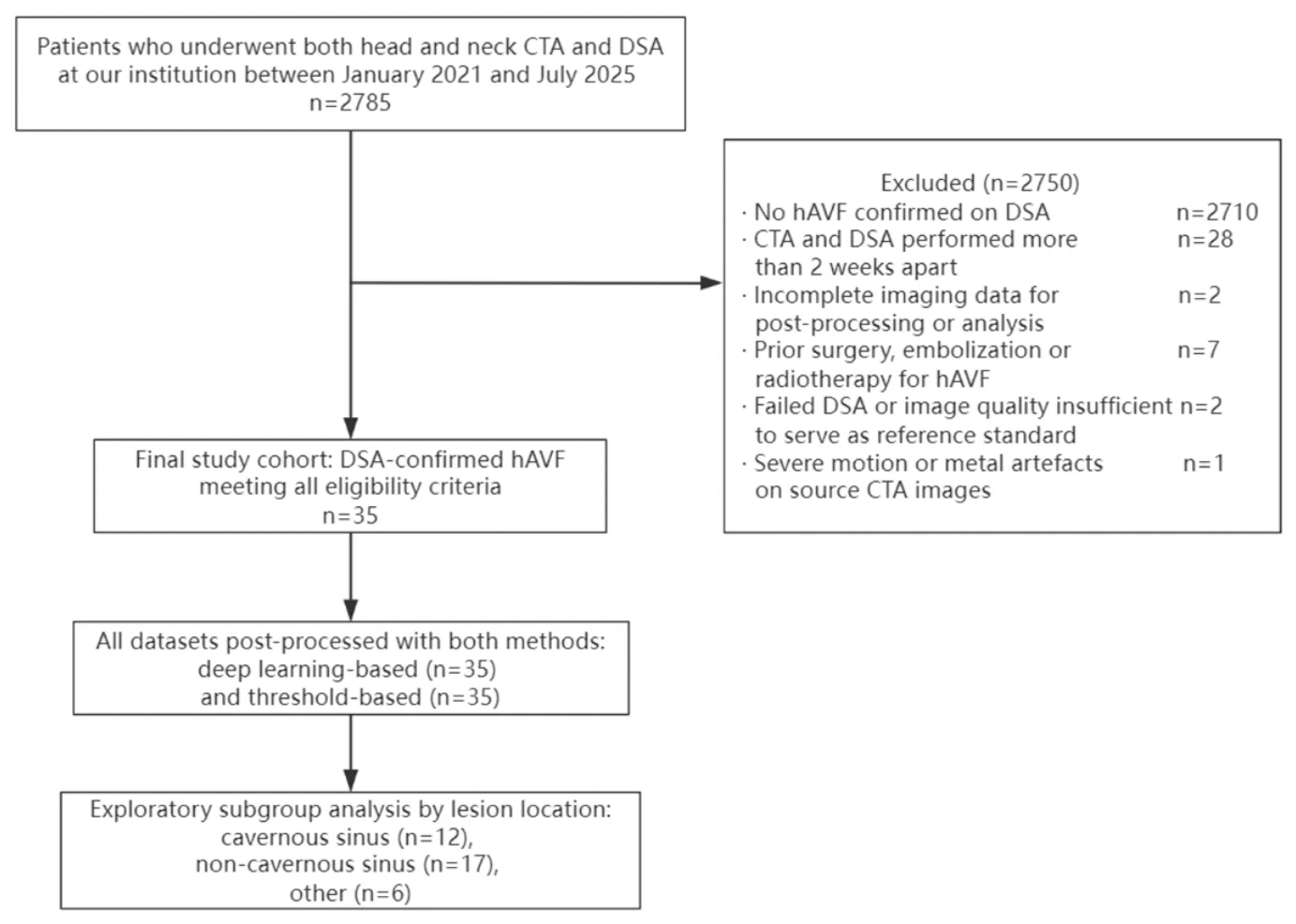
Flow diagram of patient selection. hAVF, head and neck arteriovenous fistula; CTA, computed tomography angiography; DSA, digital subtraction angiography.

Patients were eligible if they met all of the following criteria: (1) DSA-confirmed hAVF; (2) CTA and DSA performed within two weeks of each other; (3) complete imaging data available for post-processing and analysis. Patients were excluded if they had (1) prior surgery, embolization or radiotherapy for hAVF or related cerebrovascular malformations; (2) failed DSA or image quality insufficient for use as the reference standard; or (3) severe motion or metal artefacts in the source CTA images. Patient selection is summarised in Figure 1.

Because only DSA-positive cases were enrolled, this study compares the two bone-removal methods with respect to image quality, key-structure display and technical visualisation in known-positive cases; it was not designed to estimate sensitivity, specificity, predictive values or the area under the receiver operating characteristic curve, and no diagnostic accuracy measure is reported.

The study was approved by the hospital’s Institutional Review Board (approval number 2025-477-01), and informed consent was waived because of the retrospective study design.

### 2.2. CTA Acquisition Protocol

Scans were performed on either a Canon Aquilion ONE (Canon Medical Systems, Otawara, Japan) or a NeuViz Epoch (Neusoft Medical Systems, Shenyang, China) scanner. All examinations were performed after intravenous bolus injection of iodinated contrast, with arterial-phase acquisition from the aortic arch to the vertex. Reconstructed slice thickness was ≤ 1 mm, matrix 512 × 512, tube voltage 80–120 kVp, tube current 140–280 mA, rotation time 0.5 s, and acquisition collimation 0.5–0.625 mm. Iodinated contrast (iomeprol, 400 mg I/mL) was administered through a cubital vein with a power injector, using a personalised injection protocol with bolus-triggered or fixed-delay acquisition. CTA was performed on the Canon scanner in *n* = 31 patients and on the Neusoft scanner in *n* = 4 patients.

### 2.3. Deep Learning Model

The algorithm is a commercial deep learning-based bone-removal implementation (RFR-CTASub) developed by Neusoft Medical Systems and provided as a locked version on the AVW workstation (version 2.0.17.319). The deep learning-based bone-removal algorithm is built on a three-dimensional BottleNeckResUnet architecture. The network takes axial CTA volumes of 512 × 512 × 32 voxels as input. In the encoder, the initial convolution uses a 3 × 3 × 3 kernel with a stride of 2, followed by instance normalisation and LeakyReLU activation. The encoder comprises four progressive downsampling stages, with feature-channel numbers of 16, 32, 64, 80, and 96. The first stage contains three basic residual blocks, whereas the subsequent encoder stages use bottleneck residual blocks. Each bottleneck residual block consists of a 1 × 1 × 1 dimensionality-reduction convolution, a 3 × 3 × 3 convolution, and a 1 × 1 × 1 dimensionality-expansion convolution, followed by instance normalisation and LeakyReLU activation, with residual connections to facilitate feature propagation. Spatial and channel attention mechanisms are incorporated into the high-level feature representations to enhance salient anatomical features.

The decoder uses 4 × 4 × 4 transposed convolutions with a stride of 2 for progressive upsampling. Features from corresponding encoder levels are integrated through skip connections, and the decoder feature-channel numbers are 80, 64, 32, and 16. The model adopts a multitask learning strategy that jointly performs skull-suppression image regression and skull segmentation to enrich feature representation. The regression branch is optimised using a combination of Charbonnier loss and gradient-difference loss, whereas the segmentation branch uses a weighted logistic regression loss combined with focal loss (γ = 2; positive and negative sample weights of 0.25 and 0.75, respectively). A semi-supervised learning strategy is further incorporated [27,28], in which the regression and segmentation losses serve as supervised objectives, while self-aware and cross-sample-aware prototype consistency constraints serve as unsupervised objectives. The segmentation output is used to constrain the skull boundary and reduce partial-volume effects around the skull, thereby improving the quality of the resulting bone-suppressed vascular images.

None of the patients included in the present study contributed to the training, validation, or parameter tuning of the deep learning model.

### 2.4. Image Post-Processing

All contrast-enhanced CTA datasets were processed independently with both methods. For deep learning-based removal, data were uploaded to a commercial advanced visualisation workstation (AVW, version 2.0.17.319; Neusoft Medical Systems, Shenyang, China) and bone removal was performed automatically with a deep learning algorithm (RFR-CTASub). For threshold-based removal, conventional attenuation-threshold automatic bone removal was performed on the same workstation with the vendor’s fully automatic two-stage threshold-based algorithm. This algorithm applies a fixed high threshold of 450 HU to extract high-confidence bone seeds and a fixed low threshold of 200 HU to define the candidate bone region; these are the vendor’s default values and were identical for all patients. A local adaptive threshold region-growing method was then applied, followed by morphological opening, three-dimensional hole filling and largest connected component preservation.

Threshold-based bone removal is a standard and routine function for head and neck CTA post-processing on this workstation. Neither method involved manual adjustment of the attenuation threshold or manual deletion or correction of residual bone or vascular structures. The same source sequence was used for both methods. Volume rendering (VR) and maximum intensity projection (MIP) reconstructions were generated after processing, and all reconstruction parameters other than the bone-removal method were kept constant.

To assess workflow efficiency, the time required for bone-removal post-processing was recorded for each patient and each method using a stopwatch operated by the same operator. Timing started when the bone-removal procedure was initiated and stopped when the final evaluable vascular reconstruction was displayed. Both methods were performed by the same operator to limit the effect of operator experience.

### 2.5. Image Assessment

All post-processed reconstructions were evaluated independently by two radiologists with 8 and 12 years of experience in neurovascular imaging, who were blinded to the DSA results and clinical data. VR and MIP reconstructions for each case were presented together. Reading order was randomised across patients and methods, the two methods were presented in separate reading sessions separated by a wash-out interval of at least two weeks, and the readers did not discuss their scores. Readers were not informed which method had been used, and no method labels or vendor identifiers appeared on the reconstructed images.

#### 2.5.1. Subjective Image Quality

Overall image quality was scored on a 5-point scale: 5, excellent—smooth sharp vessel margins with no residual bone and fully adequate diagnostic quality; 4, good—minimal tiny residual bone not affecting vessel visualisation or diagnosis; 3, fair—partial residual bone or rough vessel margins but still diagnostic; 2, poor—substantial residual bone or vessel pseudo-defects affecting diagnostic confidence; 1, very poor—failed bone removal and non-diagnostic image.

#### 2.5.2. Vessel Edge Sharpness

Vascular edge sharpness was quantitatively assessed using the AVW workstation (version 2.0.17.319). The C1 segment of the internal carotid artery was selected for measurement. A measurement line was placed perpendicular to the vascular edge, and the workstation automatically generated a CT attenuation profile along the line. The absolute slope of the CT attenuation profile across the vascular edge was used as a quantitative measure of vascular edge sharpness. A larger absolute slope indicates a sharper vascular margin. The same measurement procedure was applied to both bone-removal methods for each CTA dataset.

#### 2.5.3. Display of Key Anatomical Structures

The fistula site, feeding artery and draining vein were scored separately on a 3-point scale, adapted from scoring systems used for key vascular structures in previous CTA studies and modified for hAVF [29]: 2, excellent—clearly displayed with sharp margins; 1, fair—identifiable but with slightly blurred contrast or margins; 0, not identifiable—the structure is obscured by bone or has been erroneously removed together with the vascular signal. The three item scores were summed to a total score ranging from 0 to 6.

Technical visualisation of the hAVF was defined as a total score greater than 0, that is, at least one of the three structures being identifiable. This is a display endpoint: it records whether the processed images preserve and show key hAVF structures. It is derived from the same three item scores as the primary endpoint, is therefore not independent evidence, and is not a diagnostic determination or a substitute for DSA. DSA served as the reference standard for hAVF diagnosis and for defining the cohort.

### 2.6. Statistical Analysis

Analysis was performed in R (version 4.4.1). Continuous variables are reported as mean ± standard deviation when approximately normally distributed and as median with interquartile range otherwise. Paired Wilcoxon signed-rank tests compared image quality and anatomical structure scores between methods. The magnitude of each difference was summarised as the mean paired difference with its 95% confidence interval. Objective vascular edge sharpness and post-processing time were compared with paired *t* tests because the paired differences were approximately normally distributed; mean paired differences and 95% confidence intervals are reported. McNemar’s test compared technical visualisation rates; because the number of discordant pairs was small, exact test results were also considered. Inter-observer agreement was assessed with linearly weighted Cohen kappa and interpreted as follows: ≤0.20 poor, 0.21 to 0.40 slight, 0.41 to 0.60 moderate, 0.61 to 0.80 good, 0.81 to 1.00 almost perfect [30]. Subgroup analyses by lesion location were exploratory; their *p* values are unadjusted for multiple testing and are interpreted descriptively. A two-sided *p* < 0.05 was considered statistically significant. Multiplicity was controlled by Bonferroni correction across the 11 pre-specified confirmatory comparisons (α = 0.05/11); raw and adjusted *p* values are reported in Appendix A.2, Table A2.

## 3. Results

### 3.1. Baseline Characteristics

Thirty-five patients with DSA-confirmed hAVF were included: 20 women (57.1%) and 15 men (42.9%), aged 15 to 74 years (mean 45.9 ± 14.8 years). By lesion location there were 17 (48.6%) non-cavernous sinus dural AVFs, 12 (34.3%) cavernous sinus dural AVFs and 6 (17.1%) other head and neck AVFs (Table 1).

### 3.2. Image Quality and Anatomical Structure Display

**Figure 2:**
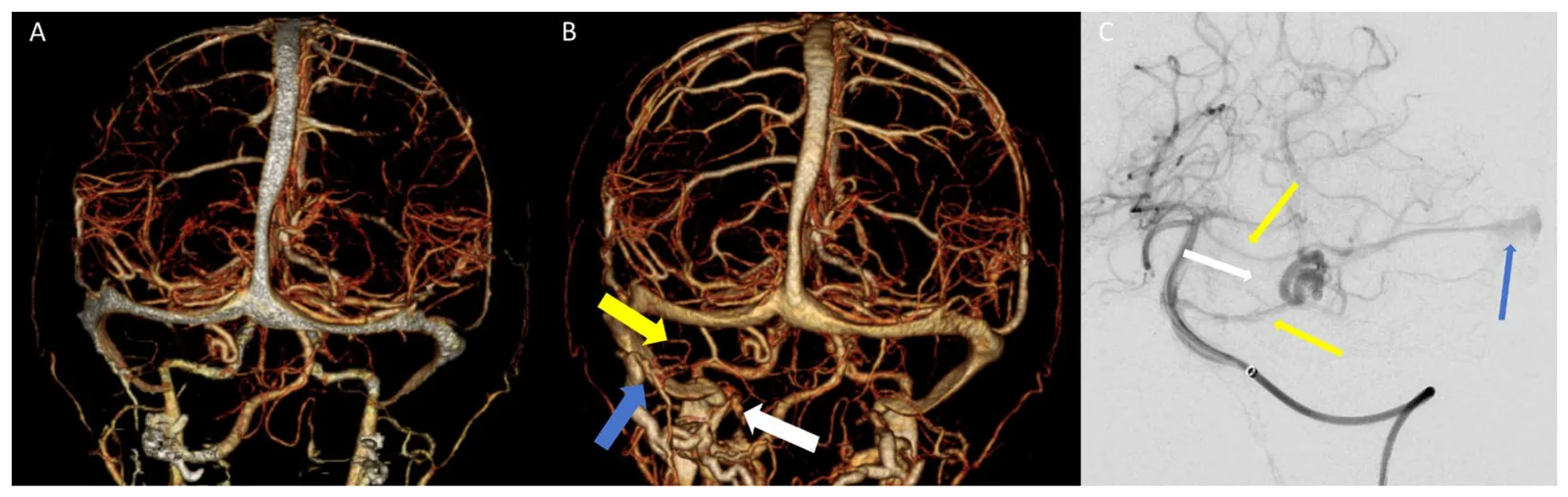
Representative CTA bone-removed VR images and DSA correlation in a head and neck arteriovenous fistula. (**A**), threshold-based bone removal. (**B**), deep learning-based bone removal (white thick arrows: fistula site at the left cerebellopontine angle; yellow thick arrows: left anterior inferior cerebellar artery and pontine artery branches; blue thick arrows: draining vein, sigmoid sinus). (**C**), DSA (white thin arrows: fistula site at the left cerebellopontine angle; yellow thin arrows: left anterior inferior cerebellar artery and pontine artery branches; blue thin arrows: draining vein, sigmoid sinus). This case is illustrative and does not necessarily represent the overall performance of either method.

**Figure 3:**
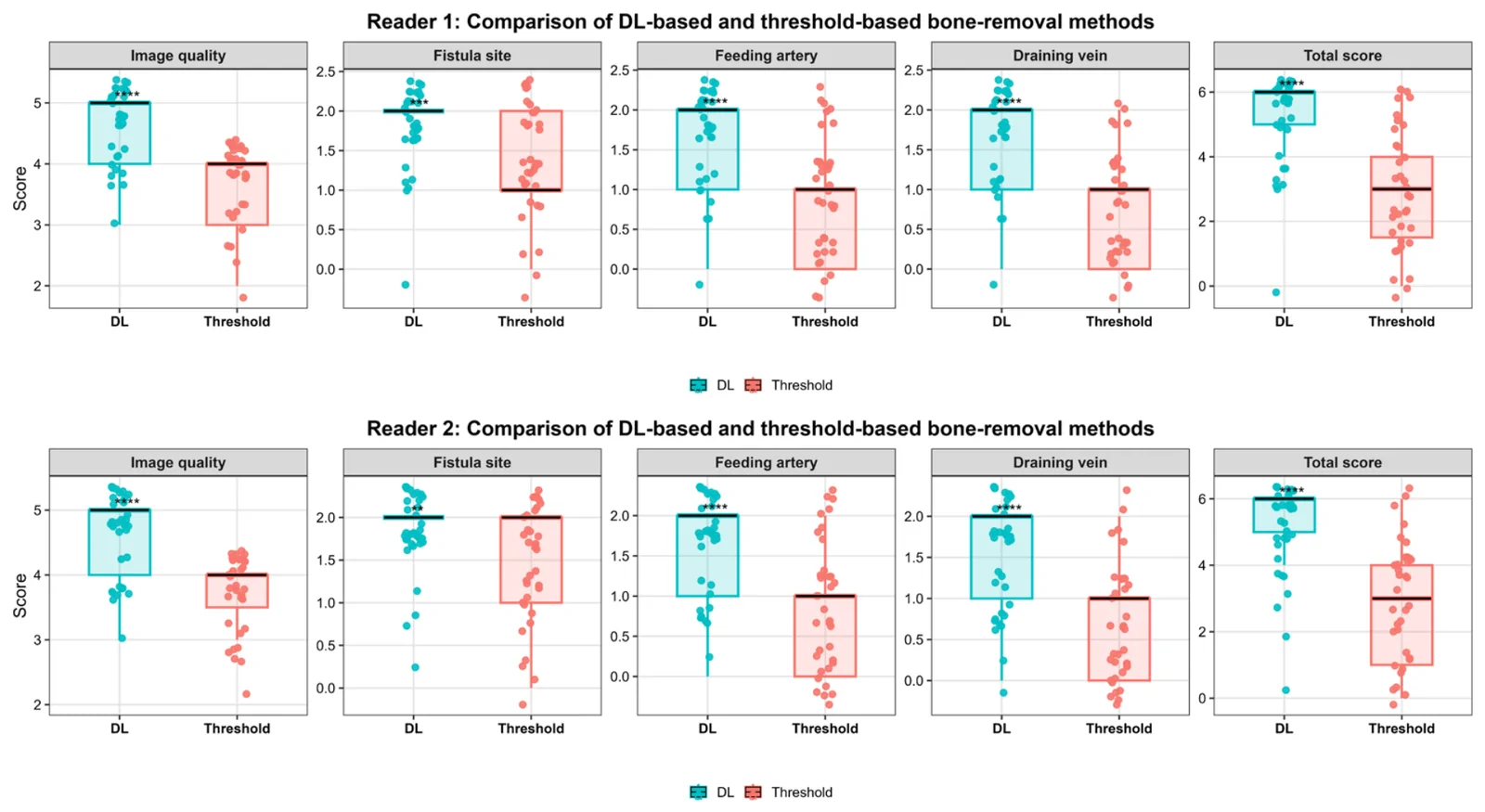
Comparison of image quality and anatomical structure scores after deep learning-based and threshold-based bone removal as assessed by the two radiologists. Boxes indicate the interquartile range, the horizontal line the median, and the whiskers the minimum and maximum observed values; light boxes denote deep learning-based removal and dark boxes threshold-based removal. The y-axis is scaled to the range of each item (1–5 for overall image quality, 0–2 for the fistula site, feeding artery and draining vein, and 0–6 for the total score), and results are shown separately for reader 1 and reader 2. Asterisks indicate statistically significant differences between the two methods (paired Wilcoxon signed-rank test): ** *p* < 0.01, *** *p* < 0.001, **** *p* < 0.0001.

Results for both readers are given in Table 2 and Figure 2 and Figure 3. Median overall image quality was 5.00 [4.00, 5.00] with deep learning-based removal versus 4.00 [3.00, 4.00] with threshold-based removal for reader 1 (mean paired difference, 1.00 [95% CI, 0.86–1.14]; *p* < 0.001), and 5.00 [4.00, 5.00] versus 4.00 [3.50, 4.00] for reader 2 (mean paired difference, 0.94 [95% CI, 0.83–1.06]; *p* < 0.001), a median difference of one scale point for both readers. Median scores for the fistula site, feeding artery and draining vein were higher with deep learning-based removal for both readers (all *p* ≤ 0.001; Table 2). Median total score was 6.00 [5.00, 6.00] versus 3.00 [1.50, 4.00] for reader 1 and 6.00 [5.00, 6.00] versus 3.00 [1.00, 4.00] for reader 2 (both *p* < 0.001). For the objective assessment of vascular edge sharpness, the absolute slope of the CT attenuation profile across the C1 segment of the internal carotid artery was significantly higher with deep learning-based removal than with threshold-based removal (316.47 ± 46.68 vs. 273.70 ± 45.66; mean paired difference, 42.77 [95% CI, 37.90–47.64]; *p* < 0.001). Figure 2 shows a representative non-cavernous sinus case in which the fistula site, feeding artery and draining vein are depicted after deep learning-based removal but partly obscured after threshold-based removal.

### 3.3. Technical Visualisation in DSA-Positive Cases

Technical visualisation was the binary form of the same item scores, defined as display of at least one of the three structures. Rates are given in Table 3. Deep learning-based removal yielded 34 of 35 (97.1%) versus 31 of 35 (88.6%) with threshold-based removal, with three discordant pairs. The risk difference was 8.6 percentage points (95% CI −4.6 to 21.7), and Cohen’s h was 0.36, indicating a small-to-moderate effect. McNemar’s test showed no statistically significant difference (*p* = 0.248). With only three discordant pairs, this comparison has very low power, and the 95% CI includes both no difference and a potentially important benefit. The endpoint is therefore inconclusive rather than negative, and we report it descriptively.

**Figure 4:**
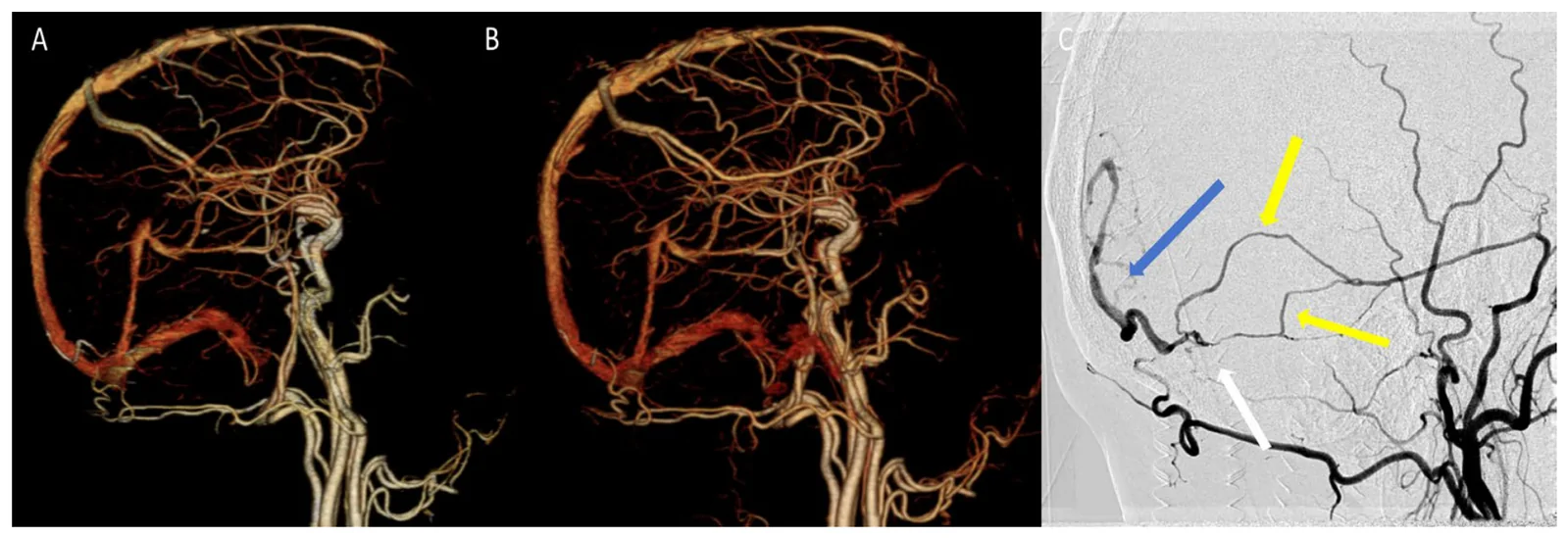
Representative case in which both bone-removal methods failed to depict the fistula: right lateral sinus dural arteriovenous fistula. (**A**), threshold-based bone removal. (**B**), deep learning-based bone removal. (**C**), DSA showing the fistula point (white arrow) supplied by middle meningeal artery branches (yellow arrow) and draining into a cortical vein (blue arrow). This case is illustrative and does not necessarily represent the overall performance of either method.

The single case not visualised by deep learning-based removal, a right lateral sinus dural AVF, was also not visualised by threshold-based removal (Figure 4). Three of the four cases not visualised by threshold-based removal were cavernous sinus lesions. Because the primary threshold may be considered liberal, we repeated the comparison under four stricter definitions of the endpoint as a robustness check. Under every stricter definition the direction of the effect was unchanged and the difference became statistically significant (Appendix A.3, Table A3). The pre-specified endpoint remains inconclusive, whereas the stricter definitions indicate that threshold-based removal more often achieved partial rather than complete display.

### 3.4. Inter-Observer Agreement

Linearly weighted Cohen’s kappa analysis showed good to almost perfect agreement between the two radiologists for image quality and key anatomical structure scores with both bone-removal methods (Table 4). For deep learning-based removal, the weighted kappa values were 0.765 (95% CI, 0.538–0.992) for overall image quality, 0.811 (95% CI, 0.544–1.000) for fistula site, 0.754 (95% CI, 0.515–0.992) for feeding artery, and 0.629 (95% CI, 0.386–0.873) for draining vein. For threshold-based removal, the corresponding values were 0.761 (95% CI, 0.547–0.975), 0.839 (95% CI, 0.684–0.994), 0.751 (95% CI, 0.585–0.918), and 0.885 (95% CI, 0.755–1.000). The weighted kappa values for total anatomical structure score were 0.786 (95% CI, 0.649–0.924) and 0.809 (95% CI, 0.702–0.916), respectively.

### 3.5. Exploratory Subgroup Analysis by Lesion Location

**Figure 5:**
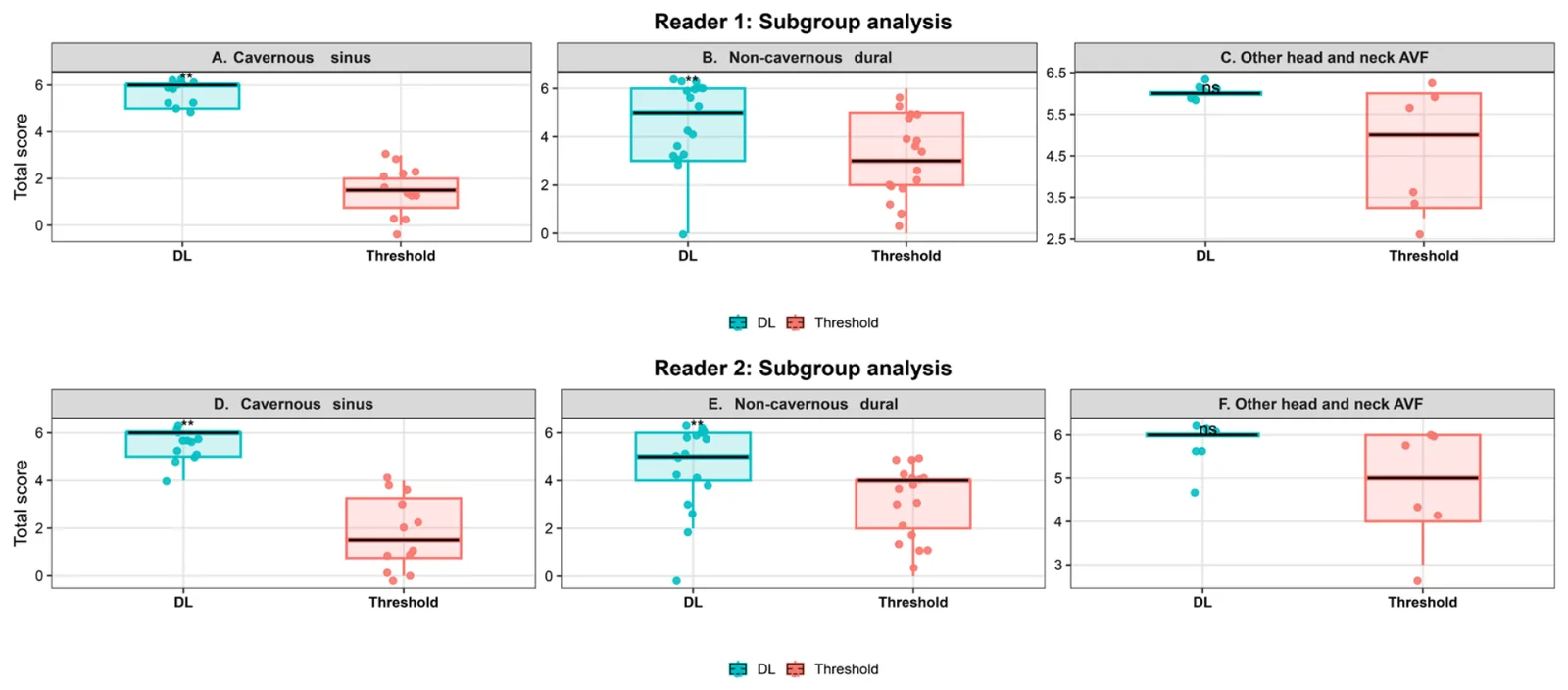
Comparison of total anatomical structure scores on CTA images after deep learning-based and threshold-based bone removal, grouped by lesion location (exploratory subgroup analysis). Boxes indicate the interquartile range, the horizontal line the median, and the whiskers the minimum and maximum observed values; light boxes denote deep learning-based removal and dark boxes threshold-based removal. The y-axis is the total anatomical structure score (0–6), shown separately for each reader and lesion-location subgroup. Asterisks indicate statistically significant differences between the two methods (paired Wilcoxon signed-rank test): ** *p* < 0.01; ns, not significant.

Results are given in Table 5 and Figure 5. In cavernous sinus dural AVF (*n* = 12), median total score with deep learning-based removal was 6.00 [5.00, 6.00] for both readers versus 1.50 [0.75, 2.00] and 1.50 [0.75, 3.25] with threshold-based removal. The corresponding mean paired differences were 4.25 (95% CI, 3.53–4.97) for reader 1 and 3.67 (95% CI, 2.54–4.79) for reader 2 (both *p* = 0.002). Technical visualisation was 12 of 12 (100.0%) versus 9 of 12 (75.0%).

In non-cavernous sinus dural AVF (*n* = 17), median total scores were 5.00 [3.00, 6.00] and 5.00 [4.00, 6.00] versus 3.00 [2.00, 5.00] and 4.00 [2.00, 4.00]. The corresponding mean paired differences were 1.35 (95% CI, 0.54–2.16) and 1.47 (95% CI, 0.64–2.31) (both *p* = 0.003). Technical visualisation was 16 of 17 (94.1%) with both methods.

**Figure 6:**
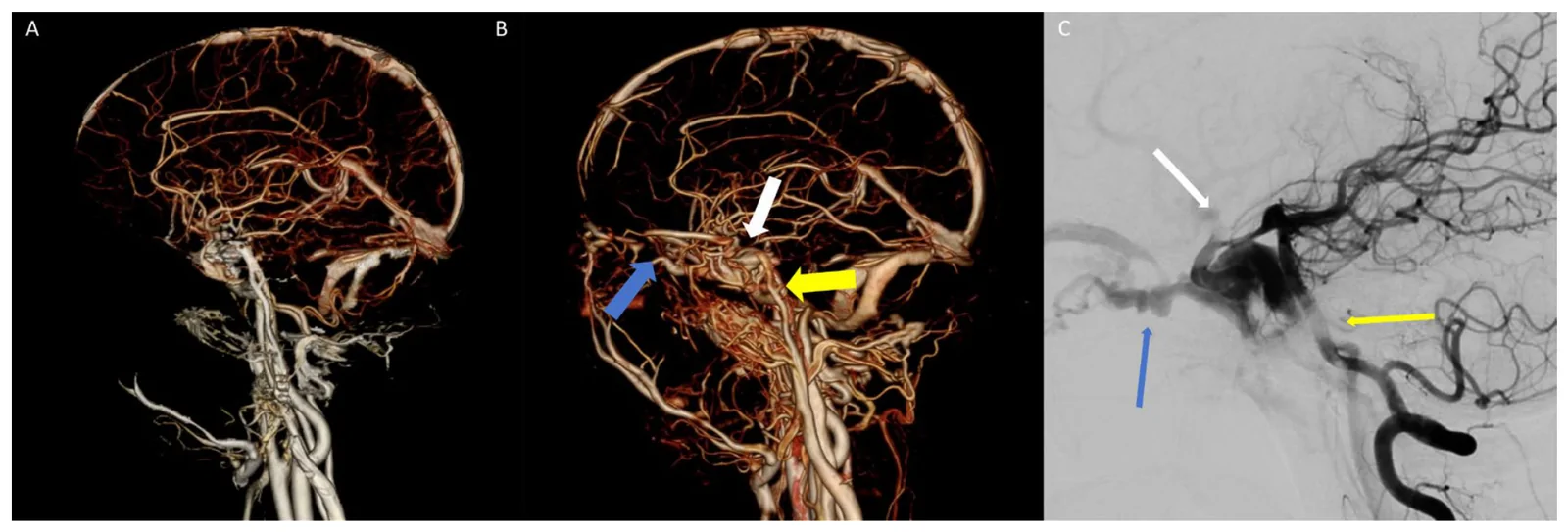
Representative CTA bone-removed VR images and DSA correlation in a cavernous sinus arteriovenous fistula. (**A**), threshold-based bone removal. (**B**), deep learning-based bone removal (white thick arrows: fistula site in the cavernous sinus; yellow thick arrows: internal carotid artery; blue thick arrows: draining vein, inferior ophthalmic vein). (**C**), DSA (white thin arrows: fistula site in the cavernous sinus; yellow thin arrows: internal carotid artery; blue thin arrows: draining vein, inferior ophthalmic vein). This case is illustrative and does not necessarily represent the overall performance of either method.

In the other hAVF group (*n* = 6), the mean paired differences were 1.33 (95% CI, −0.25–2.91) for reader 1 and 1.00 (95% CI, −0.15–2.15) for reader 2 (*p* = 0.10 and 0.08, respectively). Both methods achieved technical visualisation in all 6 cases (100.0%). Given the small subgroup sizes, these analyses were exploratory and were not adjusted for multiple comparisons. Figure 6 shows a representative cavernous sinus case for illustration.

### 3.6. Post-Processing Time

Mean post-processing time was 40.56 ± 1.61 s (median 40.54 s; range 37.62–45.76 s) with deep learning-based removal versus 101.70 ± 4.39 s (median 100.62 s; range 92.58–110.65 s) with threshold-based removal. The mean paired difference was 61.14 s (95% CI, 59.53–62.74 s; paired t test, *p* < 0.001).

## 4. Discussion

In this paired comparison, two commercially implemented bone-removal post-processors were applied to the same head and neck CTA datasets in 35 DSA-confirmed hAVFs. Deep learning-based removal displayed the fistula site, feeding artery and draining vein more completely than threshold-based removal, and the automated measurement of vessel edge sharpness was concordant. Overall image quality was higher and post-processing was about one minute shorter per case, whereas technical visualisation rates did not differ significantly. These results support one bounded claim: within a cohort of known-positive hAVFs, deep learning-based bone removal displays the structures that determine treatment planning more completely and does so faster.

The image quality advantage is mechanistically plausible. Threshold-based removal separates bone from vessel on attenuation alone, and it therefore fails precisely where the attenuation of contrast-enhanced blood overlaps that of cortical bone and calcification, and where partial volume effects are most pronounced [15]. Those are the skull base, the petrous apex and the parasellar region, which is why the threshold-based misses clustered in the cavernous sinus. A network that uses three-dimensional spatial topology rather than a single attenuation cut-off can in principle keep thin feeding arteries and perifistular communications intact while suppressing bone, which is what the higher feeding artery and draining vein scores reflect. The structures that decide whether a fistula can be treated endovascularly and by which route are the fistula site, the feeding artery origin and the drainage pattern, and these are the structures on which the score difference was largest. Whether this improved display translates into better treatment-planning accuracy, procedural success or clinical outcomes was not evaluated in the present study.

The technical visualisation result deserves restraint. The rate was numerically higher with deep learning-based removal but not significantly so, and with three discordant pairs the comparison is uninformative rather than negative. Two further points bound it. The endpoint is derived from the same three item scores and therefore provides no independent evidence; moreover, its threshold is low, because assigning a score of 1 to any single structure is sufficient. We therefore treat visualisation as a descriptive summary of display, not as accuracy. Some hAVFs remained poorly depicted after either method, which may reflect a small shunt, very thin abnormal vessels, or haemodynamic factors that a static acquisition cannot capture. As illustrated in Figure 4, both methods may fail to depict a fistula in small or low-flow lesions, and DSA remains the reference standard for definitive assessment. Deep learning-based bone removal may improve what a conventional head and neck CTA can display; it does not replace DSA for definitive assessment of fistula architecture.

The subgroup signal at the cavernous sinus is exploratory and hypothesis-generating only, and it is the least secure finding in this study. Twelve patients, three discordant cases and no multiplicity adjustment cannot support a claim beyond hypothesis generation. If real, the effect would matter because the cavernous sinus is where bone and vessel are closest and where threshold-based removal is most likely to fail. A multicentre study with negative controls is needed to establish whether the gain is a property of the anatomy, of the algorithm, or of the sample.

The time saving should be interpreted in workflow terms rather than as an isolated number. Beyond the absolute reduction of about 61 s per case, the deep learning method requires no manual threshold selection and no iterative re-running, which reduces dependence on individual operator experience and reduces the likelihood that a case needs to be reprocessed. Because the algorithm is embedded in a commercial workstation and requires no additional hardware or dedicated computing infrastructure, it is potentially accessible to centres with limited resources. The shorter post-processing time may facilitate more efficient CTA review in clinical practice, particularly when rapid image preparation is required for neurovascular assessment. The improved vascular visualisation may also reduce the time required for repeated image manipulation and help readers identify the fistula site, feeding artery, and draining vein more efficiently. We did not measure downstream consequences such as the number of repeat post-processing runs, reporting turnaround time or the need for additional non-invasive imaging before DSA; these are the endpoints at which a workflow advantage would become clinically meaningful, and they should be addressed prospectively.

Time-resolved techniques such as 4D-CTA and time-resolved MRA address the temporal dimension of shunting and perform well in low-flow or haemodynamically complex lesions, with 4D-CTA reported at 93% sensitivity and 98% specificity for intracranial shunts [31,32,33,34], but they require a dedicated acquisition. The present question is narrower and complementary: for a patient who has already had a conventional head and neck CTA, can better post-processing recover the anatomical detail needed to plan DSA? Prior deep learning bone-removal work has shown faster reconstruction [21], accurate skull segmentation [22] and better general vessel display [20,24], and one study has shown a downstream clinical detection benefit [23]. None anchored the comparison to DSA in hAVF or scored the fistula site, feeding artery and draining vein separately.

Two further perspectives qualify the findings. First, the readers in this study were diagnostic radiologists; whether the improved display translates into greater confidence in treatment planning for interventional neuroradiologists, who are the end users of these reconstructions, was not assessed. Second, the algorithm evaluated here is a single commercial implementation, and the results should not be extrapolated to deep learning bone-removal products from other vendors, whose training data and network architectures differ. Publicly accessible datasets relevant to CTA and cerebrovascular image analysis are summarised in Appendix A.4, Table A4; none provides head and neck CTA with bone-removal reference and hAVF annotation, which is why an institutional DSA-confirmed cohort was used.

This study has several limitations. First, this was a single-centre retrospective study with a modest sample size, and several subgroup analyses by lesion location were based on few cases, were exploratory and hypothesis-generating only. Because the cohort was retrospectively determined by the available eligible cases, no formal a priori sample-size calculation was performed and no subgroup was sized to detect a location-specific difference. A post hoc precision analysis (Appendix A.1, Table A1) indicated that the sample size was sufficient to detect the large effects observed for the primary paired endpoints, but remains insufficient for the binary technical visualisation endpoint and for subgroup comparisons. In addition, 31 of 35 examinations were performed on one scanner; because both methods were applied to the same source dataset, this heterogeneity does not confound the paired comparison, but the four examinations on the second scanner are too few to assess platform effects. Second, because only DSA-positive cases were enrolled, there was no negative control group, so specificity, false-positive rate, positive and negative predictive values, and the area under the receiver operating characteristic curve could not be evaluated; this is the most important limitation for readers seeking a diagnostic accuracy estimate. Third, defining technical visualisation as a total score greater than 0 reflects preservation and display of key anatomical structures; it is not equivalent to a definitive clinical diagnosis or to direct use for treatment planning. Fourth, complete blinding to processing method cannot be guaranteed: the two methods differ in the amount of residual bone, which is itself an item in the image-quality scale, so a reader could in principle infer which reconstruction was produced by which method. This would not affect the automated edge-sharpness measurement or the processing-time comparison. Future studies could extend this deep learning bone-removal technique to 4D-CTA and integrate it with standard classifications such as Borden, Cognard or Barrow, together with signs of high-risk venous drainage and cortical venous reflux. Multicentre, large-sample prospective studies with negative controls are needed to validate the stability, external applicability and actual impact on clinical decision-making of this technique.

## 5. Conclusions

In DSA-confirmed head and neck arteriovenous fistulas, deep learning-based CTA bone removal improved subjective image quality, objective vessel edge sharpness, and display of the key anatomical structures, and reduced post-processing time by about one minute per case, compared with conventional threshold-based removal. Technical visualisation rates did not differ significantly. The apparent advantage at the cavernous sinus is exploratory and requires confirmation in a larger, multicentre study that includes negative controls.

## Figures and Tables

**Table 1:** Baseline characteristics of the study subjects.

Characteristic		Statistic (*n* = 35)
Age (mean +/− SD, years)		45.9 +/− 14.8
Sex	Male	15 (42.9%)
	Female	20 (57.1%)
Lesion location/type	Non-cavernous sinus dural AVF	17 (48.6%)
	cavernous sinus dural AVF	12 (34.3%)
	Other head and neck AVF	6 (17.1%)

**Table 2:** Comparison of image quality and anatomical structure display scores between the two bone-removal methods.

Metric	Reader 1, DL-Based	Reader 1, Threshold-Based	Reader 1, *p* Value	Reader 1, Mean Paired Difference (95% CI)	Reader 2, DL-Based	Reader 2, Threshold-Based	Reader 2, *p* Value	Reader 2, Mean Paired Difference (95% CI)
Overall image quality	5.00 [4.00, 5.00]	4.00 [3.00, 4.00]	<0.001	1.00 (0.86–1.14)	5.00 [4.00, 5.00]	4.00 [3.50, 4.00]	<0.001	0.94 (0.83–1.06)
Fistula site	2.00 [2.00, 2.00]	1.00 [1.00, 2.00]	<0.001	0.49 (0.26–0.71)	2.00 [2.00, 2.00]	2.00 [1.00, 2.00]	0.001	0.43 (0.20–0.65)
Feeding artery	2.00 [1.00, 2.00]	1.00 [0.00, 1.00]	<0.001	0.86 (0.59–1.12)	2.00 [1.00, 2.00]	1.00 [0.00, 1.00]	<0.001	0.83 (0.55–1.11)
Draining vein	2.00 [1.00, 2.00]	1.00 [0.00, 1.00]	<0.001	1.00 (0.72–1.28)	2.00 [1.00, 2.00]	1.00 [0.00, 1.00]	<0.001	0.89 (0.60–1.17)
Total score	6.00 [5.00, 6.00]	3.00 [1.50, 4.00]	<0.001	2.34 (1.67–3.02)	6.00 [5.00, 6.00]	3.00 [1.00, 4.00]	<0.001	2.14 (1.48–2.80)

**Table 3:** Technical visualisation of the two bone-removal methods in DSA-positive cases.

Metric	Reader	DL-Based	Threshold-Based	Risk Difference (95% CI)	Cohen’s h	*p* Value
Visualised,n/N(%)	1	34/35 (97.1%)	31/35 (88.6%)	8.6% (−1.1–18.3)	0.36	0.248
Visualised,n/N(%)	2	34/35 (97.1%)	31/35 (88.6%)	8.6% (−1.1–18.3)	0.36	0.248

**Table 4:** Inter-observer agreement between the two radiologists.

Item	DL-Based Weighted Kappa	Threshold-Based Weighted Kappa
Overall image quality	0.765 (0.538–0.992)	0.761 (0.547–0.975)
Fistula site score	0.811 (0.544–1.000)	0.839 (0.684–0.994)
Feeding artery score	0.754 (0.515–0.992)	0.751 (0.585–0.918)
Draining vein score	0.629 (0.386–0.873)	0.885 (0.755–1.000)
Total anatomical score	0.786 (0.649–0.924)	0.809 (0.702–0.916)

**Table 5:** Exploratory subgroup analysis results by lesion location.

Group	*n*	Reader	DL-Based Total Score	Threshold-Based Total Score	Mean Paired Difference (95% CI)	*p* Value	DL-Based Visualisation Rate	Threshold-Based Visualisation Rate
Cavernous sinus dural AVF	12	Reader 1	6.00 [5.00, 6.00]	1.50 [0.75, 2.00]	4.25 (3.53–4.97)	0.002	12/12 (100.0%)	9/12 (75.0%)
		Reader 2	6.00 [5.00, 6.00]	1.50 [0.75, 3.25]	3.67 (2.54–4.79)	0.002	12/12 (100.0%)	9/12 (75.0%)
Non-cavernous sinus dural AVF	17	Reader 1	5.00 [3.00, 6.00]	3.00 [2.00, 5.00]	1.35 (0.54–2.16)	0.003	16/17 (94.1%)	16/17 (94.1%)
		Reader 2	5.00 [4.00, 6.00]	4.00 [2.00, 4.00]	1.47 (0.64–2.31)	0.003	16/17 (94.1%)	16/17 (94.1%)
Other	6	Reader 1	6.00 [6.00, 6.00]	5.00 [3.00, 6.00]	1.33 (−0.25–2.91)	0.102	6/6 (100.0%)	6/6 (100.0%)
		Reader 2	6.00 [5.75, 6.00]	5.00 [3.75, 6.00]	1.00 (−0.15–2.15)	0.083	6/6 (100.0%)	6/6 (100.0%)

## Data Availability

The imaging data presented in this study are not deposited in a public repository. The data were obtained under institutional ethics approval and are subject to patient privacy and institutional data-governance restrictions; the current ethics approval does not provide for unrestricted public deposition of the full imaging dataset. De-identified data may be made available from the corresponding author upon reasonable request, subject to institutional privacy and ethics approval.

## Abbreviations

The following abbreviations are used in this manuscript:

4D	Four-dimensional
AVF	Arteriovenous fistula
CTA	Computed tomography angiography
DL	Deep learning
hAVF	Head and neck arteriovenous fistula
MIP	Maximum intensity projection
MRA	Magnetic resonance angiography
VR	Volume rendering

## Appendix A

### Appendix A.1. Post Hoc Precision Analysis

Cohen’s dz was calculated as the mean paired difference divided by the standard deviation of the paired differences. Mean differences are deep learning-based minus threshold-based for image quality and anatomy scores; for processing time, negative values indicate time saved by deep learning-based removal. The 95% CIs are for the mean paired difference. Post hoc power was computed for a paired t test at a two-sided α = 0.05 and is reported as a supplementary precision analysis, not as a substitute for an a priori sample-size calculation. *p* values are from paired Wilcoxon signed-rank tests, except for vessel edge sharpness and processing time, which are from paired t tests. The binary technical visualisation endpoint was not included because only three discordant pairs were observed, resulting in very low power.

**Table A1:** Post hoc precision analysis for primary endpoints.

Endpoint	Cohen’s dz	Post Hoc Power	Mean Diff (95% CI)	Wilcoxon *p*
Image quality, reader 1	2.38	>0.999	1.00 (0.86–1.14)	<0.001
Image quality, reader 2	2.79	>0.999	0.94 (0.83–1.06)	<0.001
Anatomy total, reader 1	1.19	>0.999	2.34 (1.67–3.02)	<0.001
Anatomy total, reader 2	1.11	>0.999	2.14 (1.48–2.80)	<0.001
vascular edge sharpness	3.02	>0.999	42.77 (37.90–47.64)	<0.001
Processing time (s)	13.09	>0.999	61.14 (59.53–62.74)	<0.001

### Appendix A.2. Multiplicity Adjustment for the Pre-Specified Confirmatory Comparisons

All comparisons were pre-specified and hypothesis-driven. Bonferroni correction was applied across the family of 11 confirmatory comparisons (overall image quality, fistula site, feeding artery and draining vein for each of the two readers; vessel edge sharpness at the ICA C1 segment; post-processing time; and technical visualisation rate), giving a significance threshold of alpha = 0.05/11 = 0.0045. Inter-observer agreement (Cohen kappa) involves no hypothesis testing and the analysis stratified by lesion location is exploratory; neither was included. Table A2 lists raw and Bonferroni-adjusted *p* values. Every comparison that was significant before correction remained significant after correction (largest adjusted *p* = 0.014), and the technical visualisation rate was non-significant both before and after correction; multiplicity adjustment therefore changed no conclusion of the study.

**Table A2:** Raw and Bonferroni-adjusted *p* values for the 11 pre-specified comparisons.

Comparison	Raw *p*	Bonferroni-Adjusted *p*
Post-processing time (s)	<0.001	<0.001
Overall image quality, Reader 1	<0.001	<0.001
Overall image quality, Reader 2	<0.001	<0.001
Fistula site, Reader 1	<0.001	0.005
Feeding artery, Reader 1	<0.001	<0.001
Draining vein, Reader 1	<0.001	<0.001
Fistula site, Reader 2	0.001	0.014
Feeding artery, Reader 2	<0.001	<0.001
Draining vein, Reader 2	<0.001	<0.001
Vessel edge sharpness, ICA C1 (HU/mm)	<0.001	<0.001
Technical visualisation rate	0.248	1.000

### Appendix A.3. Sensitivity Analysis of the Technical Visualisation Endpoint

The primary definition of technical visualisation was pre-specified as display of at least one of the three structures (fistula site, feeding artery, draining vein), that is, a total anatomical structure score of 1 or more. Because this threshold may be considered liberal for a head and neck arteriovenous fistula, we repeated the paired comparison under four stricter, post hoc definitions. These analyses were not pre-specified and are presented as supportive rather than confirmatory; the primary endpoint is unchanged. Under every stricter definition the direction of the effect was the same and its magnitude was larger, and the comparison became statistically significant. Two findings are worth noting: display of the fistula site alone reproduced the primary definition exactly, indicating that the fistula site was the determining component in every case in this cohort; and requiring display of all three structures produced the largest difference, because threshold-based removal more often achieved partial than complete display. Risk differences and their 95% confidence intervals are based on the discordant pairs; Cohen’s h is given as the effect size for paired proportions; *p* values are from McNemar tests.

**Table A3:** Sensitivity analysis of the technical visualisation endpoint under five definitions (*n* = 35 paired datasets).

Definition of Technical Visualisation	Reader	Deep Learning-Based	Threshold-Based	Discordant Pairs (DL Only/TH Only)	Risk Difference, Percentage Points (95% CI)	Cohen’s h	*p* Value
Primary definition: any one of the three structures (total score >= 1)	1	34/35 (97.1%)	31/35 (88.6%)	3/0	8.6 (−1.1–18.3)	0.36	0.248
Primary definition: any one of the three structures (total score >= 1)	2	34/35 (97.1%)	31/35 (88.6%)	3/0	8.6 (−1.1–18.3)	0.36	0.248
Total score >= 3	1	34/35 (97.1%)	18/35 (51.4%)	16/0	45.7 (23.3–68.1)	1.20	<0.001
Total score >= 3	2	33/35 (94.3%)	21/35 (60.0%)	12/0	34.3 (14.9–53.7)	0.89	<0.001
Feeding artery or draining vein visualised	1	34/35 (97.1%)	26/35 (74.3%)	8/0	22.9 (7.0–38.7)	0.72	0.008
Feeding artery or draining vein visualised	2	34/35 (97.1%)	25/35 (71.4%)	9/0	25.7 (8.9–42.5)	0.79	0.004
Fistula site visualised (regardless of other structures)	1	34/35 (97.1%)	31/35 (88.6%)	3/0	8.6 (−1.1–18.3)	0.36	0.248
Fistula site visualised (regardless of other structures)	2	34/35 (97.1%)	31/35 (88.6%)	3/0	8.6 (−1.1–18.3)	0.36	0.248
All three structures visualised	1	34/35 (97.1%)	15/35 (42.9%)	19/0	54.3 (29.9–78.7)	1.37	<0.001
All three structures visualised	2	33/35 (94.3%)	15/35 (42.9%)	18/0	51.4 (27.7–75.2)	1.23	<0.001

### Appendix A.4. Publicly Accessible Datasets Relevant to CTA and Cerebrovascular Image Analysis

Publicly accessible datasets can facilitate the development and external evaluation of algorithms for cerebrovascular image analysis. However, datasets specifically focused on head and neck arteriovenous fistulas (hAVFs), particularly those designed for CTA bone-removal evaluation, remain limited. Table A4 summarises representative publicly accessible datasets relevant to CTA and cerebrovascular image analysis, including their imaging modalities, available annotations, cohort sizes, and access information.

**Table A4:** Publicly accessible datasets relevant to CTA and cerebrovascular image analysis.

Dataset	Imaging Modality	Task/Annotations	Number of Cases	Repository/Access
TopCoW 2024	CTA and MRA	Circle of Willis anatomical segmentation; multiclass vascular segmentation and topology-aware annotations	250 images (125 paired CTA/MRA cases) in the 2024 training release	Zenodo; open use
Large IA Segmentation dataset	3D CTA	Intracranial aneurysm segmentation; voxel-wise aneurysm annotations	1338 internal CTA cases and 138 external CTA cases	Zenodo; research-use access by request
RSNA Intracranial Aneurysm Detection (RSNA-ICA)	CTA, MRA and MRI (T1 post-contrast, T2); multimodal	Intracranial aneurysm detection and localisation at 13 arterial locations; vessel segmentations for a subset of cases	>4000 CT brain scans	AWS Open Data (S3, no account required); non-commercial licence, no redistribution
